# Estimating the Beginning of Pregnancy in German Claims Data: Development of an Algorithm With a Focus on the Expected Delivery Date

**DOI:** 10.3389/fpubh.2020.00350

**Published:** 2020-08-12

**Authors:** Tania Schink, Nadine Wentzell, Katarina Dathe, Marlies Onken, Ulrike Haug

**Affiliations:** ^1^Department of Clinical Epidemiology, Leibniz Institute for Prevention Research and Epidemiology—BIPS, Bremen, Germany; ^2^Charité – Universitätsmedizin Berlin, corporate member of Freie Universität Berlin, Humboldt-Universität zu Berlin, and Berlin Institute of Health, Institut für Klinische Pharmakologie und Toxikologie, Pharmakovigilanz- und Beratungszentrum für Embryonaltoxikologie, Berlin, Germany; ^3^Faculty of Human and Health Sciences, University of Bremen, Bremen, Germany

**Keywords:** electronic healthcare data, claims data, gestational age (GA), duration of pregnancy, beginning of pregnancy, drug safety

## Abstract

**Background:** Estimating the beginning of pregnancy is crucial when studying drug safety in pregnancy, but important information in this regard, such as the last menstrual period (LMP), is generally not recorded in claims databases. The beginning of pregnancy is therefore usually estimated by subtracting a median length of pregnancy from the date of birth. Due to the variability in pregnancy lengths, this might result in non-negligible errors. German claims data may offer the possibility to estimate the beginning of pregnancy more precisely based on the expected delivery date (EDD) which can be coded once or more often during a pregnancy.

**Purpose:** To estimate the beginning of pregnancy in German claims data focusing on the potential of the expected delivery date (EDD).

**Methods:** We included data of all pregnancies in women aged 12–50 years ending in a live birth between 2006 and 2015 identified in the German Pharmacoepidemiological Research Database (GePaRD). We assessed the number of coded EDDs per pregnancy and the concordance if ≥ 2 EDDs were coded. We estimated the beginning of pregnancy by subtracting 280 days from the EDD or the most frequent EDD (in case of discordant EDDs). To examine plausibility, we determined the distribution of pregnancy lengths and assessed whether the gestational age at which prenatal examinations were coded was plausible. For pregnancies without EDD, the beginning was estimated by subtracting the respective observed median lengths of pregnancy for preterm births, term births, and births after due date from the actual dates of birth.

**Results:** In 82.4% of pregnancies, at least one EDD was available (thereof 6.1% with only one EDD and 80.9% with ≥ 2 EDDs that were all concordant). The maximal difference between discordant EDDs was in median 5 days (interquartile range: 3–7 days). Based on the EDD, the median length of pregnancy was 276 days for term births and in 84.7% of pregnancies the second antibody screening test was performed in the recommended interval ± 2 weeks. In pregnancies without EDD the respective proportion was 84.9%.

**Conclusions:** By using the EDD, the beginning of pregnancy can plausibly be estimated in German claims data.

## Introduction

Electronic health care databases offer great potential for investigating drug utilization and drug safety in pregnancy. They avoid recall and non-response bias and—due to the typically large sample size—allow investigating the effect of rare drug exposures (1). A key prerequisite for research on drug safety in pregnancy with such databases is an appropriate algorithm to estimate the beginning of pregnancy which is crucial for specifying the gestational window vulnerable for substance-specific developmental toxicity. However, essential information in this regard, such as the last menstrual period (LMP), is generally not recorded in claims databases. Thus, the beginning of pregnancy is often estimated by subtracting the assumed average length of pregnancy from the date of birth, using different lengths for preterm, term, and post-term births (2, 3).

German claims data may offer the possibility to estimate the beginning of pregnancy more precisely given that in the coding system it is possible to record the expected delivery date (EDD). The EDD is usually calculated by adding 280 days to the date of the LMP or determined based on ultrasound measurements in early pregnancy. The EDD may be coded once or more often during a pregnancy in German claims data but may also be missing, as coding is not mandatory. If an EDD is available in claims data, the beginning of pregnancy may then be estimated by doing the reverse calculation as the gynecologist, i.e., by subtracting the 280 days—the biologically expected duration of pregnancy—from the EDD.

However, the extent of availability as well as the consistency and plausibility of information on the EDD in German claims data have not been investigated so far. Our study therefore aimed to (1) shed light on the availability, consistency and plausibility of the EDD in German claims data and (2) to develop an algorithm to estimate the beginning of pregnancy in German claims data considering the information on the EDD.

## Methods

### Data Source

We used data from the German Pharmacoepidemiological Research Database (GePaRD) from 2005 until 2015. GePaRD is based on claims data from four statutory health insurance providers in Germany and currently includes information on about 25 million persons who have been insured with one of the participating providers since 2004 or later. Per data year, there is information on ~17% of the general population, and all geographical regions of Germany are represented. In addition to demographic data, GePaRD contains information on drug dispensations, outpatient and inpatient services and diagnoses. In terms of pregnancies, the database contains information on the EDD, which may be coded once or more often per quarter in the outpatient setting. Each entry contains two parts of information (i) the expected date of delivery and (ii) the quarter and year in which the EDD was coded. The database also contains the actual date of birth and specific codes with which to identify preterm births and births occurring after the due date (4). Prenatal examinations, which are typically conducted by gynecologists in Germany, may be identified based on the respective codes of the Doctors' Fee Scale within the Statutory Health Insurance Scheme (Einheitlicher Bewertungsmaßstab, EBM), and the exact dates of examinations are available as well.

In Germany, the utilization of health insurance data for scientific research is regulated by the Code of Social Law. All involved health insurance providers as well as the German Federal (Social) Insurance Office and the Senator for Science, Health, and Consumer Protection in Bremen as their responsible authorities approved the use of GePaRD data for this study. Informed consent for studies based on GePaRD is not required by law and according to the Ethics Committee of the University of Bremen these studies are exempt from institutional review board review.

### Pregnancies

We included data of all pregnancies ending in live births between 2006 and 2015 in women of childbearing age (12–50 years) who were continuously insured with one of the statutory health insurance providers contributing data to GePaRD in the three quarters before and the quarter of the birth. The latter inclusion criterion ensured a sufficiently long observation period to obtain all available information over the full course of pregnancy. Pregnancies ending in live births were identified based on a previously developed algorithm (4) and the respective births were classified as “preterm birth,” “birth after due date,” and “term birth” (i.e., a birth not coded as preterm or after due date).

### Assessing the Availability and Consistency of Information on the EDD

For each live birth, we searched for EDDs coded in the three quarters before and the quarter of birth. We disregarded EDDs that were obviously implausible according to the following criteria: (i) the EDD was earlier than the first day of the quarter in which the EDD was coded; (ii) the EDD was more than 1 year later than the last day of the quarter in which it was coded; (iii) the actual date of birth was more than 24 weeks earlier than the EDD (i.e., a gestational age of the live birth of more than 16 weeks) or more than 3 weeks later than the EDD (i.e., after gestational week 43); (iv) the EDD was coded in the quarter of the birth and identical to the actual date of birth as it might have been coded when the actual date of birth was known, e.g., after birth or when a planned C-section was scheduled.

For the remaining EDDs, we determined the number of EDDs coded per pregnancy. If two or more EDDs were coded, we determined the proportion of pregnancies where all EDDs were concordant (i.e., shared the same date). For pregnancies with at least two discordant EDDs, we calculated the median, the 25- and 75%-quantiles (interquartile range) and the 5- and 95%-quantiles of the maximum differences between discordant EDDs.

### Using the EDD to Estimate the Beginning of Pregnancy

For pregnancies with only one EDD or pregnancies where all EDDs were concordant, the beginning of pregnancy was estimated by subtracting 280 days—the biologically expected duration of a pregnancy—from the EDD. This reverses the calculation done by the physician to get the EDD from the LMP or ultrasound examinations of the embryo.

For pregnancies with at least two discordant EDDs, we first determined the mode of EDDs, i.e., the EDD that was most often coded. If there was more than one mode, the mean of the modes was used. The latter also applies to scenarios where only discordant EDDs were coded (i.e., each EDD is then considered as a mode). We then proceeded as described above to estimate the beginning of pregnancy.

### Assessing the Plausibility of the Information on the EDD

To assess the plausibility of the information on the EDD, we examined the difference between the EDD and the actual date of birth. We stratified these analyses in preterm births, term births, and births after due date, expecting the highest agreement for term births. Furthermore, we calculated the length of pregnancy for all pregnancies with information on the EDD to assess the plausibility of the distribution. We conducted this analysis separately for births classified as preterm births, term births, and births after due date.

Finally, we assessed whether the gestational age at which certain prenatal examinations were performed was plausible if the beginning of pregnancy was estimated based on the EDD. For that purpose, we considered the first code indicating a pregnancy examination, expecting that in the majority of pregnancies, there is a code within 4 weeks after a missed menstrual bleeding (i.e., between day 28 and 56 after LMP) but not implausibly early (i.e., before potential conception or nidation). We also considered the second antibody screening which is recommended between pregnancy week 24 and 27 in Germany (5) excluding pregnancies with only one antibody screening test from this analysis. The respective codes are shown in Additional File 1.

### Estimating the Beginning for Pregnancies Without Information on the EDD

For pregnancies without information on the EDD, we estimated the beginning with the “median length method” as described and validated by Margulis et al. (2). This method subtracts 273 days from the date of birth for term births and 245 days for preterm births. It was developed based on the median (clinical) gestational age at birth among 286,432 newborns in British Columbia. As this study did not consider births after due date, we used 280 days for pregnancies ending after due date. To assess the plausibility of the estimated beginning based on this median length method we determined the gestational age at which certain prenatal examinations were performed as described above. Furthermore, in pregnancies with available EDD, we compared the beginning estimated by the median length method and by the EDD method.

## Results

We identified 1,018,310 pregnancies fulfilling the inclusion criteria. Of those, 6.8% were classified as preterm births, 79.3% as term births, and 13.9% as births after due date.

In 82.4% of pregnancies, at least one EDD fulfilling the plausibility criteria was available. In most of these pregnancies (80.9%), two or more EDDs which were all concordant were coded. In 6.1% only one EDD was identified and in 13.1% two or more EDDs which were not all concordant were identified. The maximal difference between the discordant EDDs was in median 5 days (25–75%: 3–7 days, 5–95%: 1–16 days).

**Figure 1:**
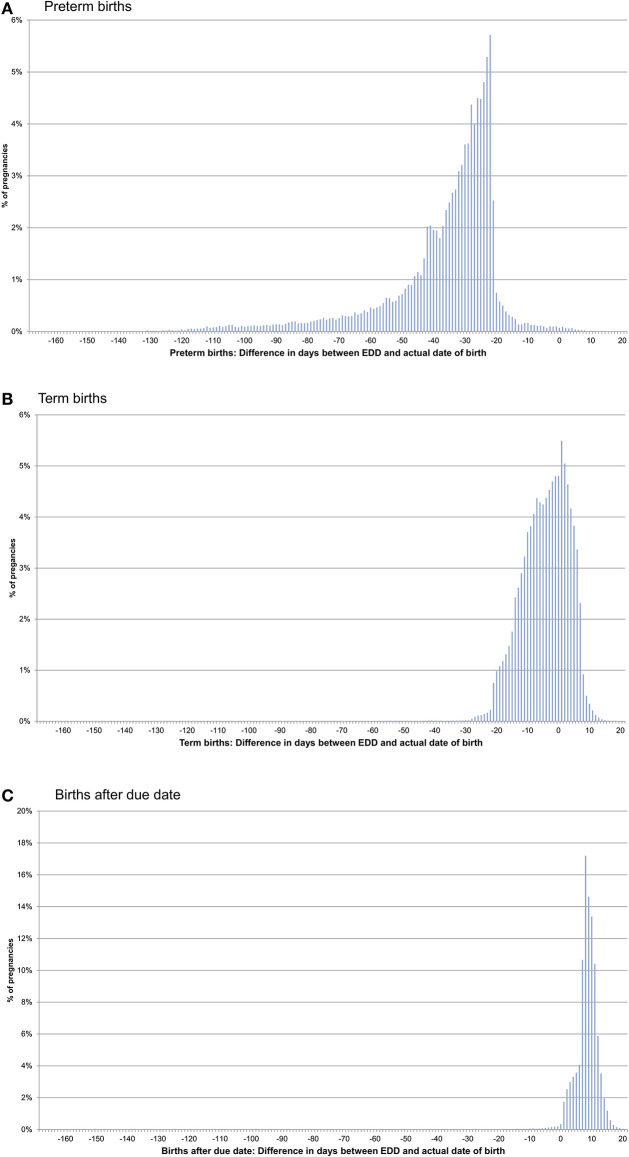
Difference between the date of birth and the expected delivery date (EDD) in pregnancies with only concordant EDDs [preterm births **(A)**, term births **(B)**, and births after due date **(C)**].

In pregnancies with only concordant EDDs, for preterm births, the actual date of birth was in median 31 days earlier than the EDD. For term births, the actual date of birth was in median 4 days earlier than the EDD and in births after due date, it was in median 9 days later (Figures 1A–C, Table 1). In pregnancies with only one EDD, the median differences were −35, −3, and +9 days for preterm births, term births, and births after due date, respectively. In pregnancies with at least two discordant EDDs, the median differences were −32, −4, and +8 days, respectively (Table 1).

**Table 1:** Difference between the expected delivery date (EDD) and the date of birth.

	**Preterm**	**Term**	**After due date**
**≥2, All concordant**	*n*= 45,015	*n*= 538,414	*n*= 94,906
Median (Q1; Q3)	−31 (−42; −25)	−4 (−9; 2)	9 (7; 10)
10% quantile; 90% quantile	−61; −22	−15; 5	4; 12
5% quantile; 95% quantile	−78; −20	−18; 6	2; 13
**Only 1 EDD**	*n*= 4,363	*n*= 39,608	*n*= 6,850
Median (Q1; Q3)	−35 (−55; −26)	−3 (−10; 1)	9 (7; 11)
10% quantile; 90% quantile	−91; −22	−16; 5	3; 13
5% quantile; 95% quantile	−107; −20	−19; 7	1; 14
**≥2, Not all concordant**^†^	*n*= 8,146	*n*= 58,862	*n*= 15,553
Median (Q1; Q3)	−32 (−43; −25)	−4 (−10; 2)	8 (6; 11)
10% quantile; 90% quantile	−64; −22	−15; 5	3; 13
5% quantile; 95% quantile	−81; −19	−19; 7	1; 14

† *For pregnancies with at least two discordant EDDs the mode or, if more than one mode, the mean of the modes was used to estimate the beginning*.

With the EDD method, the length of pregnancy was plausible, with a median of 249 days for preterm births, 276 days for term births, and 289 days for births after due date (Table 2).

**Table 2:** Length of pregnancy in days based on the EED method.

	**Median**	**Quantile 25–75%**	**Quantile 5–95%**
Preterm births	249	238–255	198–260
Term births	276	270–282	262–286
Births after due date	289	287–290	282–293

**Figure 2:**
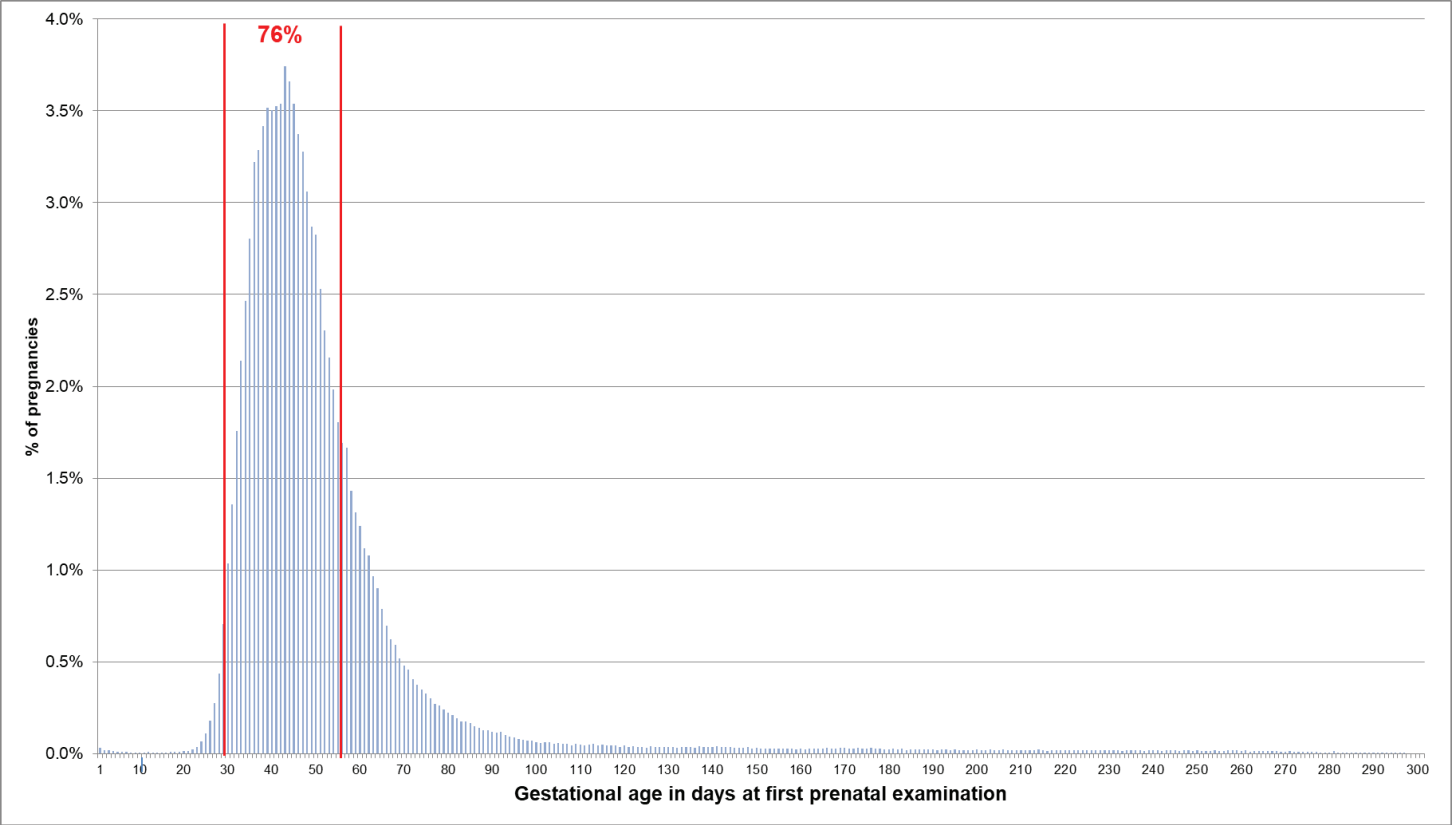
Gestational age at coding of first prenatal examination when the beginning of pregnancy is estimated based on the EDD. Bars represent percentages of pregnancies with the respective gestational age at the first prenatal examination. Red lines mark the interval between day 28 and 56 after LMP, where 76% of pregnancies had the first prenatal examination.

**Figure 3:**
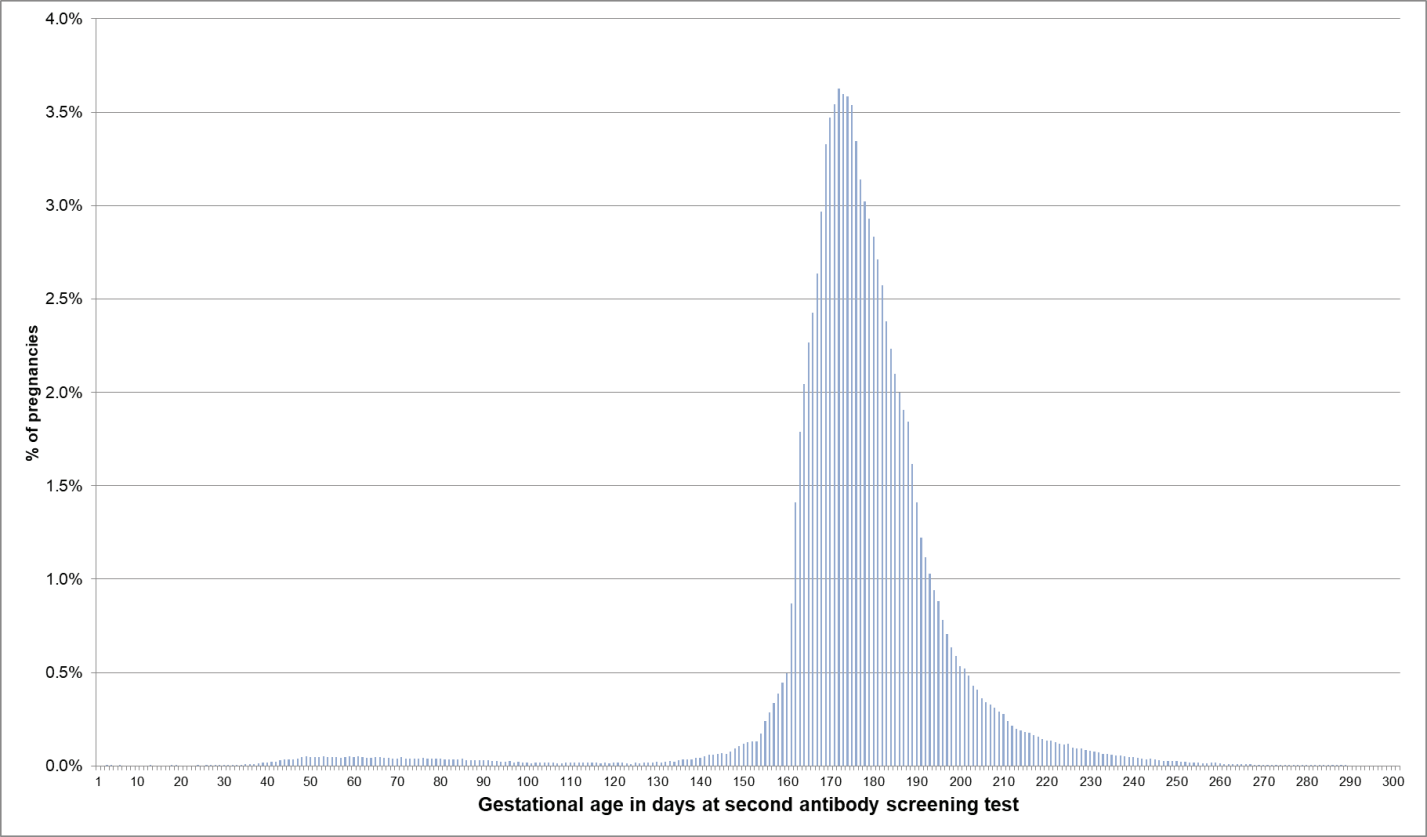
Gestational age at coding of the second antibody screening when the beginning of pregnancy is estimated based on the EDD. Bars represent percentages of pregnancies with the respective gestational age at the second antibody screening. Red lines mark the recommended interval for the screening between week 24 and 27, where 74% of the pregnancies included in this analysis had their second antibody screening.

If the EDD was used to estimate the beginning of pregnancy, 76.0% of pregnancies had a first prenatal examination between day 28 and 56 after LMP. In 0.2% of pregnancies, there was already a code before day 20 and in 0.1% before day 8 (Figure 2). In 74.1% of the 720,061 pregnancies included in the analyses regarding the antibody screening test, the test was performed in the recommended interval (i.e., 24–27 weeks). In 2.4% the test was performed in the week before and in 8.2% in the week after the recommended interval (Figure 3).

**Figure 4:**
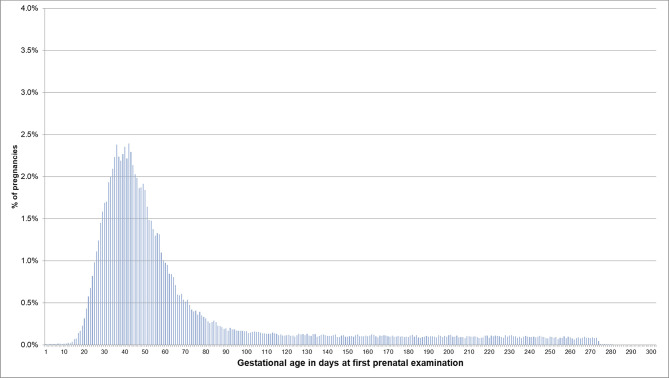
Gestational age at coding of first prenatal examination when the beginning of pregnancy is estimated based on the median length method in pregnancies with no EDD. Bars represent percentages of pregnancies with the respective gestational age at the first prenatal examination. Red lines mark the interval between day 28 and 56 after LMP, where 54% of pregnancies had the first prenatal examination.

**Figure 5:**
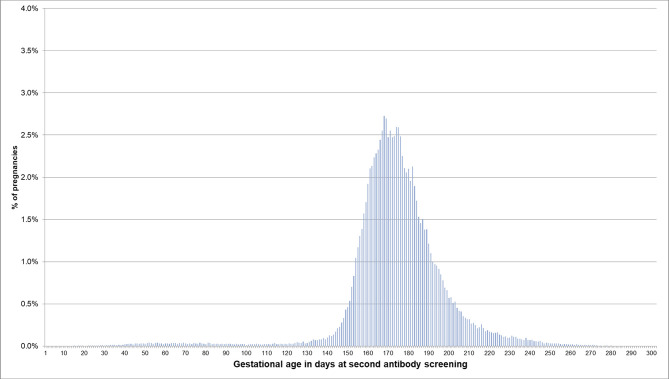
Gestational age at coding of the second antibody screening when the beginning of pregnancy is estimated based on the median length method in pregnancies with no EDD. Bars represent percentages of pregnancies with the respective gestational age at the second antibody screening. Red lines mark the recommended interval for the screening between week 24 and 27, where 61% of the pregnancies included in this analysis had their second antibody screening.

For pregnancies where the beginning of pregnancy was estimated based on the median length method because no EDDs were available, the respective distributions are shown in Figures 4, 5. In 53.5% of these pregnancies, the first prenatal examinations were performed between day 28 and 56 after LMP; 1.2% of examinations were performed before day 20, and 0.1% before day 8. In 61.3% of the pregnancies included in the analyses regarding the antibody screening test, the test was performed in the recommended interval (i.e., 24–27 weeks). In 10.1% the test was performed in the week before the recommended interval and in 7.5% in the week after the recommended interval.

**Figure 6:**
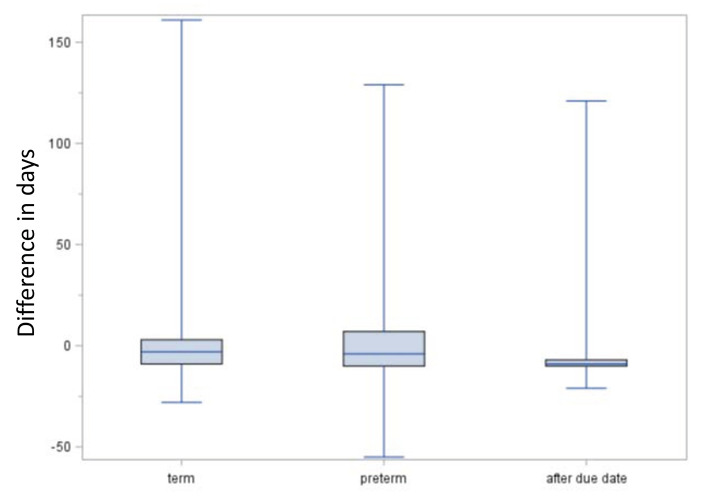
Comparison of EDD and median length method regarding the estimated date of beginning of pregnancy. Boxes indicate the upper and lower quartiles with the median in between and the ends of the whiskers mark the maximum and minimum. Negative lengths indicate an earlier estimated beginning by the expected delivery date (EDD) method compared to the median length method.

Figure 6 shows the comparison of the EDD method and the median length method regarding the estimated beginning of pregnancy. In term births, the median difference between the estimates of the EDD method and the median length method was −3 days (interquartile range: 25–75%: −9 to 3 days, 5–95%: −13 to 11 days), i.e., the estimated beginning was in median 3 days earlier with the EDD method. For preterm births, the median difference was −4 days (25–75%: −10 to 7 days, 5–95%:−15 to 47 days), and for births after due date, it was 9 days (25–75%: −10 to (−7) days, 5–95%: (−13) to 2 days).

Table 3 summarizes the algorithm based on the findings above to estimate the beginning of pregnancy in German claims data. In view of the differences between the median lengths of pregnancies used by Margulis et al. and the median lengths based on the EDD method in our study, the final algorithm uses the latter for pregnancies without recorded EDDs as the results better reflect the pregnancy lengths in the German setting. As expected, this modification led to a decrease regarding the proportion with a first prenatal examination before day 20 (0.5 vs. 1.2%).

**Table 3:** Summary of the final algorithm to estimate the beginning of pregnancy based on German claims data.

1. Search for expected delivery dates (EDDs) and selection of EDDs fulfilling the plausibility criteria:
(i) Actual date of birth not more than 24 weeks earlier and not more than 3 weeks later than the EDD^†^ (ii) EDD not before the first day of the quarter in which it was coded and not later than a year after the last day of the quarter in which it was coded (iii) If EDD coded in the quarter of the birth: EDD ≠ actual date of birth^†^^,^ ^‡^
2. Estimation of beginning of pregnancy for pregnancies with ≥ 1 plausible EDD:
(i) EDD minus 280 days if only 1 EDD or ≥ 2 EDDs that are all concordant (ii) Mode of EDDs minus 280 days if at least 2 discordant EDDs with one mode (iii) Mean of modes of EDD minus 280 days if at least 2 discordant EDDs with more than one mode
3. Estimation of beginning of pregnancy for pregnancies with no (plausible) EDD^†^:
(i) Date of birth minus 276 days if birth classified as term birth (ii) Date of birth minus 249 days if birth classified as preterm birth (iii) Date of birth minus 289 days if birth classified as birth after due date

† *These criteria are not applicable for pregnancies not ending in a live birth*.

‡ *An EDD coded in the quarter of birth that is identical to the date of birth is not implausible per se, but the EDD might have been coded when the actual date of birth was known, e.g., after birth or when a planned C-section was scheduled (see methods section)*.

## Discussion

Based on more than one million pregnancies, we developed an algorithm to estimate the beginning of pregnancy in German claims data, using the expected delivery date—which is available for more than 80% of pregnancies—as a key component. With various approaches to indirectly validate this algorithm, we found that it yields very plausible results regarding the estimated beginning of pregnancy.

In a first step, we assessed the consistency and plausibility of the information on the EDD. Regarding consistency, all EDDs were concordant in more than 80% of pregnancies and in 90% of the pregnancies with at least two discordant EDDs, the maximal difference did not exceed 16 days. To assess plausibility, we determined the differences between the EDD and the actual date of birth. As expected for live births classified as preterm, the actual date of birth was (depending on the number of concordant EDDs) in median 31–35 days earlier than the EDD. Differences of <21 days, which were observed in <5%, might either be explained by an erroneous classification of a term birth as preterm (e.g., if an impending preterm birth was prevented) or by a too early clinical estimate for the beginning. For births classified as after due date, the actual date of birth was in median 9 days later than the EDD and in only 2.7% was it more than 2 weeks later than the EDD, the point in time when labor is induced in Germany at the latest. For term births, i.e., births not classified as preterm or after due date, 90% occurred between 19 days before to 7 days after the EDD. This is well in line with nationwide data from Germany, where 91% of births occurred between gestational week 37 and 41 (6).

Furthermore, we assessed whether the gestational age at which certain prenatal examinations were performed was plausible if the beginning of pregnancy was estimated based on the EDD. We found that in 76% of pregnancies, the first prenatal examination was between week 5 and completed week 8. In 0.2% of pregnancies, it was before assumed nidation (approx. at day 20 after LMP) and in 0.1% even before conception (approx. at day 14 after LMP) (7). This, however, might be explained by prior suspected pregnancies. Overall, 85% of the second antibody screening tests were performed in the recommended interval plus/minus 1 week. Deviations from this recommended interval do not necessarily indicate an implausible EDD but could have been caused by variations in scheduling the examination. The analyses based on the median length method also showed plausible results for the estimated beginning of pregnancy, but variability and the number of less plausible results were slightly higher.

In a second step, we assessed whether the EDD method yielded plausible results regarding the distribution of pregnancy lengths. Overall, we observed a median pregnancy length of 277 days (25–75%: 270–284 days) which is a bit shorter than the 282 days (25–75%: 275–288 days) observed by Bergsjø et al. (8) based on Swedish data from 1976 to 1980, where information on the LMP was available. However, the fact that the Swedish study was conducted 40 years ago and considered only singleton pregnancies hampers the comparison to our study (8). A community-based study from the US based on data of the years 2000–2004 found a median length of 276 days and was thus rather similar to the length observed in our study (9).

The distribution of pregnancy lengths illustrates the advantage of estimating the beginning of pregnancy based on the EDD rather than assuming a fixed length of pregnancy. In our study, for example, even in the category term birth, only 69% of pregnancy lengths were ± 1 week around the median length and 10% were more than 10 days shorter than the median length. The duration of a pregnancy depends on various factors, such as age and parity of the mother, pregnancy complications, number of fetuses, and mode of delivery (8, 9). However, even within relatively homogenous groups variability is high. In Bergsjö's study, for example, among first-time mothers aged 20–34 years with singleton pregnancies ending in vaginal delivery, nearly 32% of pregnancies were either 2 weeks shorter or 2 weeks longer than the observed median length. Thus, subtracting a fixed length of pregnancy from the date of birth to estimate the beginning of pregnancy inevitably results in non-negligible errors. These might lead to relevant misclassification of exposure time when studying drug safety in pregnancy. This may be especially critical for preterm births where the individual lengths typically show high variability. Nevertheless, several databases have no other option than using a fixed length to estimate the beginning of pregnancy. Margulis et al. (3) tried to optimize and validate this method based on 286,432 mother-baby pairs. They found a 1-week agreement between the estimated and the clinical gestational age at birth in 76% of term births and in 68% of preterm births.

The advantage of the EDD for the estimation of the beginning of pregnancy is the fact that it is based on the LMP or on ultrasound measurements which are considered reliable methods to determine gestational age (10). In our database, the beginning of pregnancy could be estimated based on the EDD for more than 80% of pregnancies, while for the remaining pregnancies, the algorithm used the median length method. In future studies on drug safety in pregnancy, we will address this issue by conducting sensitivity analyses, i.e., including and excluding pregnancies where the beginning was estimated with the median length method.

As many other studies in this field using claims data we focused on live births in our analyses. However, pregnancies ending in spontaneous or induced abortions are also important for the study of drug effects during pregnancy. Apart from the fact that these pregnancies are generally under-recorded in claims data, it is particularly problematic to estimate their beginning based on the median length method. In this regard, our algorithm that is mainly based on the EDD will also offer advantages. Furthermore, the coding of the EDD might provide an opportunity (in addition to diagnosis and procedure codes) to better capture such pregnancies which we will explore in future analyses.

A limitation of studies based on GePaRD is the fact that chart validation is not possible due to data protection regulations. However, we consider it likely that the EDD determined by the physician and noted in the patient chart is identical to the EDD reported to the health insurance.

The estimation of the beginning of pregnancy is the last step in establishing GePaRD as a data source for studies on the utilization and safety of drugs during pregnancy. We already developed procedures to reliably identify pregnancies and classify their outcomes (4, 11) and to link mothers to the newborns (12). With this algorithm, we are now, for example, able to examine, in how many pregnancies the unborn child is exposed to a potentially teratogenic drug during the time window that is critical regarding exposure to the respective drug, to assess the outcome of these pregnancies, and to study the (long-term) effect of the exposure in the children.

## Conclusion

Our study suggests that the EDD offers a very good opportunity to reliably estimate the beginning of pregnancy in German claims data. It is available in more than 80% of pregnancies ending in a live birth and showed high consistency and plausibility. The algorithm developed in this study is an essential prerequisite to investigate drug safety in pregnancy based on German claims data as it allows determining the gestational age at the time of drug exposure.

## Data Availability Statement

The datasets for this study cannot be made publicly available because of data protection and legal reasons. In Germany, the utilization of health insurance data for scientific research is regulated by the Code of Social Law. Researchers have to obtain approval from the health insurance providers as well as their responsible authorities. As this approval is given only for a specific research question for a specific time and for a specific group of researches, data cannot be made publicly available.

## Ethics Statement

In Germany, the utilisation of health insurance data for scientific research is regulated by the Code of Social Law. All involved health insurance providers as well as the German Federal Office for Social Security and the Senator for Health, Women and Consumer Protection in Bremen as their responsible authorities approved the use of GePaRD data for this study. Informed consent for studies based on claims data is required by law unless obtaining consent appears unacceptable and would bias results, which was the case in this study. According to the Ethics Committee of the University of Bremen studies based on GePaRD are exempt from institutional review board review.

## Author Contributions

TS and NW: study concept and design, analysis, and interpretation of data, drafting of the manuscript, critical revision of manuscript. UH: study concept and design, interpretation of data, drafting of the manuscript, critical revision of manuscript. KD and MO: critical revision of manuscript. All authors read and approved the final manuscript.

## Conflict of Interest

The authors declare that the research was conducted in the absence of any commercial or financial relationships that could be construed as a potential conflict of interest.

## Abbreviations

EBM: einheitlicher Bewertungsmaßstab (Doctors' Fee Scale within the Statutory Health Insurance Scheme)
EDD: estimated delivery date
GePaRD: German Pharmacoepidemiological Research Database
LMP: last menstrual period.
