# Corrigendum: Estimating the Beginning of Pregnancy in German Claims Data: Development of an Algorithm With a Focus on the Expected Delivery Date

**DOI:** 10.3389/fpubh.2020.605076

**Published:** 2020-10-29

**Authors:** Tania Schink, Nadine Wentzell, Katarina Dathe, Marlies Onken, Ulrike Haug

**Affiliations:** ^1^Department of Clinical Epidemiology, Leibniz Institute for Prevention Research and Epidemiology—BIPS, Bremen, Germany; ^2^Charité – Universitätsmedizin Berlin, corporate member of Freie Universität Berlin, Humboldt-Universität zu Berlin, and Berlin Institute of Health, Institut für Klinische Pharmakologie und Toxikologie, Pharmakovigilanz- und Beratungszentrum für Embryonaltoxikologie, Berlin, Germany; ^3^Faculty of Human and Health Sciences, University of Bremen, Bremen, Germany

**Keywords:** electronic healthcare data, claims data, gestational age (GA), duration of pregnancy, beginning of pregnancy, drug safety

In the published article, there was an error in affiliations **2 and 3**. Instead of “^2^ Charité—Universitätsmedizin Berlin, corporate member of Freie Universität Berlin, Humboldt-Universität zu Berlin, Berlin, Germany, ^3^ Berlin Institute of Health, Pharmakovigilanz- und Beratungszentrum für Embryonaltoxikologie, Institut für Klinische Pharmakologie und Toxikologie, Berlin, Germany,” it should be “^2^ Charité – Universitätsmedizin Berlin, corporate member of Freie Universität Berlin, Humboldt-Universität zu Berlin, and Berlin Institute of Health, Institut für Klinische Pharmakologie und Toxikologie, Pharmakovigilanz- und Beratungszentrum für Embryonaltoxikologie, Berlin, Germany.”

The authors apologize for this error and state that this does not change the scientific conclusions of the article in any way. The original article has been updated.

