# Supplementary material for: Estimating the Beginning of Pregnancy in German Claims Data: Development of an Algorithm With a Focus on the Expected Delivery Date
Source: Front Public Health. 2020 Aug 12;8:350. doi: 10.3389/fpubh.2020.00350 (PMC7434962; doi:10.3389/fpubh.2020.00350)
Supplement: Supplementary file 1 [file Table_1.docx]

**Additional file 1:** EBM codes used for identification of the first pregnancy examination and the second antibody screening

| EBM Code since 2006 | | EBM Code in 2005 | Description |
| --- | --- | --- | --- |
| Codes used for identification of the first pregnancy examination | | | |
| 01770 | | 0100 | Care for a pregnant woman according to the German maternity guidelines of the Federal Joint Committee (G-BA) |
| 01800 | | 0124 | Test for treponemal pallidum (syphilis) using TPHA-Test in the context of prenatal care |
| 01802 | | 0125 | Rubella antibody verification using a haemolysis-in-gel (HIG) test for rubella antibodies or immunoassay if the haemagglutination inhibition titre is low (1:<32) in the context of prenatal care |
| 01804 | | 0128 | Blood type test – test of blood type (A, B, 0) and for Rh-factor D in the context of prenatal care |
| 01807 | | 0131 | Antibody screening test in the context of prenatal care |
| 01810 | | 0134 | Test for hepatitis B virus antigen (HBs-Ag) in a pregnant women in the context of prenatal care |
| 01811 | | 0135 | Test for HIV antibodies in a pregnant woman via immunoassay in the context of prenatal care |
| 01776 | | n/a | Pretest for gestational diabetes according to paragraph A nr. 8 of the German maternity guidelines of the Federal Joint Committee (G-BA) |
| 01777 | | n/a | Oral glucose tolerance test (oGTT) for gestational diabetes according to paragraph A nr. 8 of the German maternity guidelines of the Federal Joint Committee (G-BA) |
| 01812 (only for EDDs from 2013) | | n/a | Glucose assessment in venous plasma for screening for gestational diabetes according to paragraph A nr. 8 of the German maternity guidelines of the Federal Joint Committee (G-BA) |
| 01785 | | 0117 | Tocography before 28 weeks of pregnancy according to paragraph B nr. 4b of the German maternity guidelines of the Federal Joint Committee (G-BA) |
| Code used for identification of the second antibody screening test | | | |
| 01807 | 0131 | | Antibody screening test in the context of prenatal care |
